# The link in Linking

**DOI:** 10.1016/s0972-6292(16)30629-5

**Published:** 2013-06-25

**Authors:** Jane C Caldwell, Pablo A Chiale, Mario D Gonzalez, Adrian Baranchuk

**Affiliations:** 1 Heart Rhythm Service, Queen's University, Kingston, Ontario, Canada; 2 Centro de Arritmias Cardiacas de la Ciudad Autonoma de Buenos Aires, Division Cardiologia, Hospital General de Agudos J.M. Ramos Mejia, Buenos Aires, Argentina; 3 Penn State Hershey Heart and Vascular Institute, Hershey, Pennsylvania, USA

**Keywords:** Bundle Branch Block, Linking

## Abstract

We present 2 cases of the slow-fast form of AVNRT with initially narrow QRS complexes followed by sudden unexpected transition to persistently wide QRS complexes due to aberrant intraventricular conduction. Introduction of a properly timed extrastimulus in one case and critical oscillations in cycle length due to short-long coupling in the second case set the stage for the initial bundle branch block. However, persistence of the aberrancy pattern once the initial event abated was maintained by the "linking" phenomenon. Delayed, retrograde concealed activation from the contralateral bundle branch perpetuated the initial bundle branch block.

## Case Presentations

**Figure 1:**
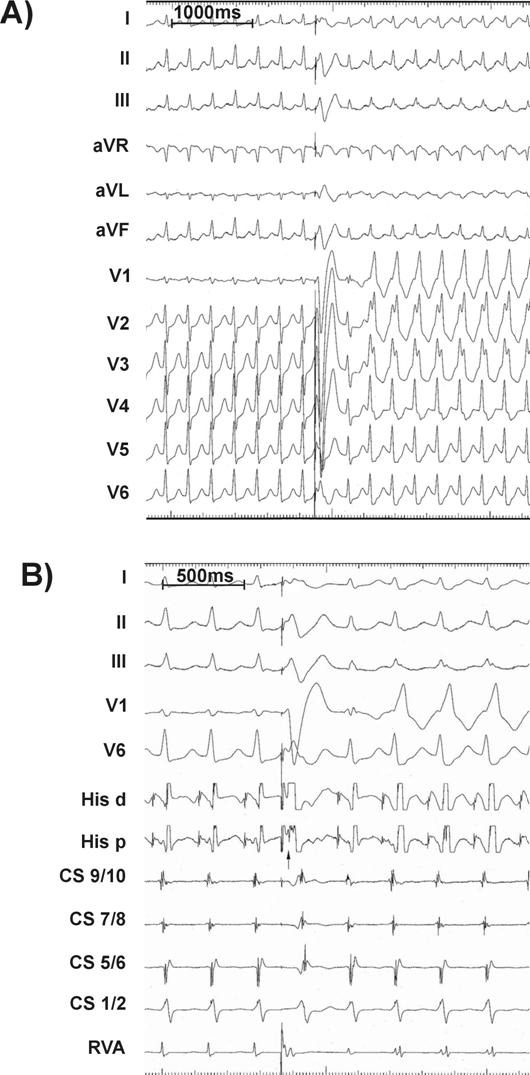
Slow-fast AVNRT with cycle length of 285 ms that was easily induced in case 1. A single premature extrastimulus (arrow in Panel B) was delivered from the right ventricular apex during AVNRT. Panel A shows the resulting persistent change in surface ECG morphology from narrow complex to RBBB. Panel B displays simultaneous intracardiac recordings which highlight the uninterrupted continuance of AVNRT with altered and delayed RV activation accompanying the development of RBBB morphology. (RVA - right ventricular apex, CS - coronary sinus, His p - His proximal dipole, His d - His distal dipole).

***Case 1:*** A 43 yr old woman with recurrent rapid palpitations but no significant past medical history and normal physical exam, resting ECG, transthoracic echocardiogram, and exercise stress test, underwent an electrophysiological study. During the procedure, several episodes of slow-fast AV nodal reentrant tachycardia (AVNRT) were induced with cycle length ~ 285ms as shown in Figure 1A. A single right ventricular (RV) premature ventricular extrastimulus converted the narrow complex tachycardia (NCT) into right bundle branch block (RBBB) morphology after an initial incomplete RBBB beat. The tachycardia cycle length remained unchanged. What is the mechanism of RBBB?

**Figure 2:**
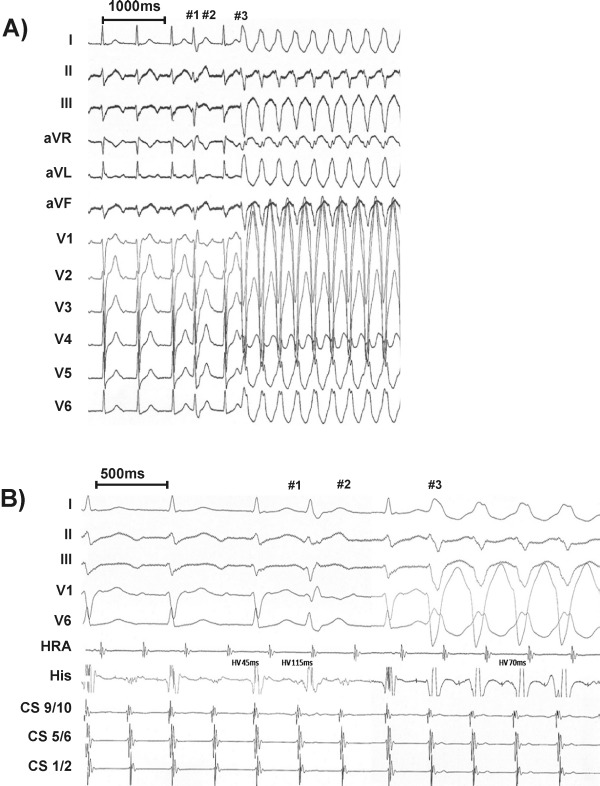
Slow-fast AVNRT was induced in case 2. In panel A, the surface ECG shows narrow QRS complexes during 2:1 conduction. Unexpected conduction with right bundle branch block (#1) was associated with a long H-V interval (115 ms) indicating delayed activation of the His bundle or left bundle branch. After another blocked beat (#2), the tachycardia was associated with 1:1 conduction with LBBB aberrancy pattern (#3). Panel B displays simultaneous intracardiac recordings which again highlight the uninterrupted continuance of AVNRT. (HRA - high right atrium, CS - coronary sinus, His - His distal dipole).

***Case 2:***: A 50 yr old man presented with recurrent effort-related, rapid palpitations but with no significant past medical history, normal physical examination, resting ECG, transthoracic echocardiogram and exercise stress test. There was an episode of sustained regular narrow QRS tachycardia lasting one hour during a 24 hr Holter. During the electrophysiology study, typical AVNRT (cycle length 300 ms) was induced. Figure 2A shows the simultaneous 12 leads ECG recording during tachycardia. On this occasion, initially the AVNRT is maintained with 2:1 A-V conduction to the ventricles with narrow QRS complexes. Unexpectedly, transient 1:1 A-V conduction to the ventricles occurs (fourth QRS complex) with a prolonged H-V interval (115 ms) and RBBB morphology, indicating bilateral slow conduction. After another 2:1 sequence, one to one conduction is initiated with firstly narrow followed by persistent left bundle branch block (LBBB). What is the mechanism of LBBB?

## Commentary

In both cases typical narrow complex AVNRT transforms into persistent BBB morphology following an ipsilateral BBB beat.

In case 1, the premature RV depolarization retrogradely activated the right bundle as demonstrated by the earlier retrograde His bundle activation (arrow). This premature activation of the right bundle branch (Figure 1B), with a short-long sequence produced a prolongation of the right bundle refractoriness [1] with the result that the next QRS shows an incomplete right bundle branch block. After that, block in the RBB is perpetuated by repeated retrograde invasion by the impulses coming from the left bundle, the so called "linking phenomenon" [2,3]. In this concept, it is the delayed retrograde activation of the bundle that extends its refractory period and thus perpetuates the antegrade bundle block. This has been demonstrated in accessory A-V pathways [4].

In case 2, during the 2:1 AV conduction a premature beat with a RBBB pattern occurs which is most likely due to aberrant conduction in the RBB (with a long HV interval indicating slow conduction in the other bundle). Right bundle branch block is the most frequently observed aberrancy in premature supraventricular beats [1] and the beat fulfils the Ashman phenomenon "long-short coupling interval" rule of aberrancy [2]. With this in mind, one can assume that the LBB is activated first by this premature beat, but what is occurring at the same instance in the RBB? If the impulse completely fails to depolarize the RBB, and there was no "concealed" retrograde invasion of the RBB, the subsequent diastolic interval of the RBB will be prolonged, thus prolonging its action potential, its refractoriness and facilitating RBB aberrancy when 1:1 AV conduction occurs. However, this is not the case, as the 1:1 conduction has LBBB aberrancy. This implies that the RBB has been invaded retrogradely by the "premature" impulse via antegrade activation of the LBB ("linking"). Thus the RBB's next diastolic interval would be shorter than that of the LBB, promote a shorter refractory period and thus permit conduction via the RBB whilst block would occur in the still refractory LBB (i.e. LBBB aberrancy). By comparison, the cycle length sequence experienced by the LBB would be (i) "short" in the premature LBB beat (ii) "long" between the premature LBB beat and the subsequent SVT beat, and then (iii) "short" in the second beat of the 1:1 AV conduction run. This sequence of "short-long-short" cycle lengths, previously described by Denker et al1 and termed the "Akhtar phenomenon" by Rosenbaum et al [3], explains the mechanism of LBBB aberrancy that was then perpetuated by a "linking" mechanism.

In summary, these cases demonstrate that different initial events can give rise to functional bundle branch block during AVNRT. In one case, a properly applied ventricular extrastimulus preexcited one bundle branch that subsequently blocked antegradely, while in the other case oscillations in cycle length with short-long sequence gave rise to the first QRS with bundle branch block. However, perpetuation of block was not due to the initial event, but rather was secondary to active interaction with the contralateral bundle branch, such that concealed, delayed retrograde activation from the contralateral bundle branch acted to maintain the functional antegrade block; a phenomenon known as "linking" phenomenon [2-4].
